# Natural human postural oscillations enhance the empathic response to a facial pain expression in a virtual character

**DOI:** 10.1038/s41598-021-91710-5

**Published:** 2021-06-14

**Authors:** Thomas Treal, Philip L. Jackson, Jean Jeuvrey, Nicolas Vignais, Aurore Meugnot

**Affiliations:** 1 CIAMS, Université Paris-Saclay, 91405 Orsay, France; 2 CIAMS, Université D’Orléans, 45067 Orléans, France; 3 École de Psychologie, Université Laval, Québec, Canada; 4 Centre Interdisciplinaire de Recherche en Réadaptation Et Intégration Sociale (CIRRIS), Québec, Canada; 5 CERVO Research Center, Québec, Canada

**Keywords:** Psychology, Empathy, Perception

## Abstract

Virtual reality platforms producing interactive and highly realistic characters are being used more and more as a research tool in social and affective neuroscience to better capture both the dynamics of emotion communication and the unintentional and automatic nature of emotional processes. While idle motion (i.e., non-communicative movements) is commonly used to create behavioural realism, its use to enhance the perception of emotion expressed by a virtual character is critically lacking. This study examined the influence of naturalistic (i.e., based on human motion capture) idle motion on two aspects (the perception of other’s pain and affective reaction) of an empathic response towards pain expressed by a virtual character. In two experiments, 32 and 34 healthy young adults were presented video clips of a virtual character displaying a facial expression of pain while its body was either static (still condition) or animated with natural postural oscillations (idle condition). The participants in Experiment 1 rated the facial pain expression of the virtual human as more intense, and those in Experiment 2 reported being more touched by its pain expression in the idle condition compared to the still condition, indicating a greater empathic response towards the virtual human’s pain in the presence of natural postural oscillations. These findings are discussed in relation to the models of empathy and biological motion processing. Future investigations will help determine to what extent such naturalistic idle motion could be a key ingredient in enhancing the anthropomorphism of a virtual human and making its emotion appear more genuine.

## Introduction

Do you remember how you reacted when you saw a street artist mimicking a stone statue for the first time? Perhaps you tried to detect blinking or small variations in their head or body posture that would help you differentiate between a human or a stone statue? These subtle movements are usually ignored in social interactions, except in this type of situation where the humanness of the person is ambiguous. In particular, this anecdote illustrates how much these non-communicative movements—coined *idle motion* by Egges and collaborators^1^—may be a core ingredient when animating a virtual character that is talking or displaying a dynamic facial expression, and that is being used as a research tool to study emotion communication in humans (i.e., emotion recognition, empathy, emotional contagion, etc.). The ability of virtual characters to create an ecological interaction while maintaining high control of the interaction is well accepted^1–4^. The current challenge is not necessarily to create a fully interactive virtual character, but one that is realistic enough for participants to accept that it might behave like a real person, such as feeling and expressing emotions. Indeed, its emotion must be believable and provoke a spontaneous and socially expected reaction from participants in order to capture the unintentional and automatic nature of emotional processes^2^.

However, a virtual character with a fixed body may be perceived spontaneously as a fictional character rather than a virtual social agent. Inconsistency between a fixed body and a dynamic face could trigger a sense of eeriness towards the virtual agent and hinder interaction with it. Thus, *idle motion* is generally implemented by default on virtual characters to increase their behavioural realism^5^ or to make the character seem ‘alive’ in a neutral (control) condition without introducing confusing factors^6–8^. Idle motion includes a “varied range of features such as subtle variations or shift in body posture caused by small muscle contraction, eye movements or blinking, breathing”^1,9^. For instance, in Groom and colleagues^5^, the conversational agent “blinked and moved her eyes in a natural fashion”. In another study^7^, the agent in the control condition of Experiment 2 “displays a neutral respiration”. In the study of Krämer and colleagues^8^, “the virtual human did not show any kind of feedback but merely slight and randomised posture shifts and eye blinking in order to appear alive (idle behavior)”. Surprisingly, except for one study^10^, no empirical evidence supports that these subtle movements influence the perception of emotions expressed by a virtual character. Treal and collaborators^10^ demonstrated that the facial pain expression of a virtual character was perceived as more intense and more believable when a subtle to-and-fro movement mimicking human dynamic equilibrium (postural oscillations) was added to its trunk, compared with a static condition. Although the method was artificial (i.e., the movement was mechanical and manually implemented in the animation software), these results prompted further exploration into how much idle motion might contribute to make the facial expressions of a virtual person more believable.

Such work is of great interest given the growing number of platforms using virtual humans as a therapeutic tool for clinical populations with social cognition deficits^11^, or in serious games to improve clinical reasoning skills in healthcare professionals^12–15^. For instance, by improving a virtual character’s anthropomorphism, a higher affective reaction may be elicited towards its pain, which may be desirable in order to train healthcare professionals in emotional regulation. Emotion regulation is one of the three components of empathy, a critical skill in medical care, which has been defined as the ability to share and understand others’ emotion or thinking, and feel motivated to help^16^. Empathy is also underlaid by two other main processes^17–21^: (1) *perspective-taking*, which refers to explicit reasoning about others’ mental states; and (2) an automatic and emotional component called *affective resonance,* which allows an individual to represent what the other might feel based on one’s own, similar, affective experiences. While the underlying processes of empathy are well documented in the field of affective neuroscience^20,22,23^, the literature is far from clear on its behavioural manifestation, i.e. how to describe and assess the empathetic behaviour resulting from the combination of these three components^24^. So far, empathy for pain has been assessed primarily using pain intensity judgment tasks, since it was implicitly, yet rightly assumed that the perception (or evaluation) of another’s pain would be influenced by the way one would empathise with this person. However, being empathetic is not restricted to a perceptual (or cognitive) dimension. Indeed, it may cover a broad spectrum of mental states and aspects of a behaviour^22^. Importantly, empathy would provoke an affective (or emotional) reaction, i.e. a sense of mixed emotions that generally accompanies the way one perceives another’s emotion, e.g., distress, sympathy or compassion when seeing pain in others^16,25–27^. The above-mentioned mechanism of emotional regulation is then paramount to contain one's own emotional reaction to avoid being overwhelmed by others’ emotions^18,28^. However, it can be challenging for health professionals to both maintain compassionate care and cope with self-oriented unpleasant emotions provoked by emotionally distressing situations^29–31^. Thus, platforms using virtual patients may be an innovative tool to help caregivers better recognise their own emotional reactions and develop strategies enabling them to reduce emotional contamination.

The present study aimed to examine the impact of idle motion in increasing the humanness of a virtual agent by using a new and ecological paradigm based on human motion capture to reproduce natural postural oscillations in a virtual character. This method captured the complex pattern of body movements reflecting postural oscillations, and in particular, included respiratory movements that are closely linked to postural control^32^. While in past studies, idle motion has usually been generated using algorithms^1,9^ or a hybrid system combining motion capture data with procedural motion generation^33^, to our knowledge, applying unconstrained, natural human postural oscillations to a virtual character paired with dynamic facial expression has not been previously examined. Yet, it has been shown that biological motion improved the virtual character’s acceptability^3,34,35^. In line with these works, it was assumed that biological motion would be a suitable method for idle animation. Especially, this study investigated whether these subtle movements (i.e., natural postural oscillations) would enhance the humanness of the virtual character, and thus significantly influence participants’ empathic response towards its facial pain expression. As pointed out, empathetic behaviour is by nature elusive and difficult to assess. Thus, in the present study, the term *empathic response* referred to two distinct, yet interacting, facets of an empathetic behaviour: the *perception* of *others’ pain* and the *affective reaction to others’ pain*. These facets were assessed through two experiments: in Experiment 1, which was based on traditional experimental paradigms of pain empathy, the participants rated the intensity of a dynamic facial pain expression in a virtual character whose body was or was not animated with natural postural oscillations. It was expected that they would perceive the virtual character’s pain as more intense in the *idle condition* compared with the *still condition*. Using the same materials, Experiment 2 aimed to assess the affective reaction that spontaneously stems from the perception of other’s pain and is posited to be part of the empathic response. The objective of Experiment 2 was then to determine whether participants would report a greater affective reaction when seeing a virtual character’s pain expression in the presence of idle motion, compared with a still condition. It was predicted that participants would report being touched more by the character’s pain expression when its body was animated with natural postural oscillations (*idle condition*) than when it was in the *still condition*.

## Experiment 1

### Method

#### Participants

Thirty-two young adults from the Faculty of Sport Science of Paris-Saclay University took part in the experiment (mean age = 18.31 years, SD = 0.53, range 18–19 years, 16 women). The sample size was based on a previous study with a similar design^10^. Each participant had normal or corrected-to-normal vision. The study was approved by the Ethics Committee of Paris-Saclay University (#CER-Paris-Saclay-2020-147) and the participants gave written informed consent to take part in this study. The experiment was conducted in accordance with the Declaration of Helsinki.

#### Material and tasks

**Figure 1:**
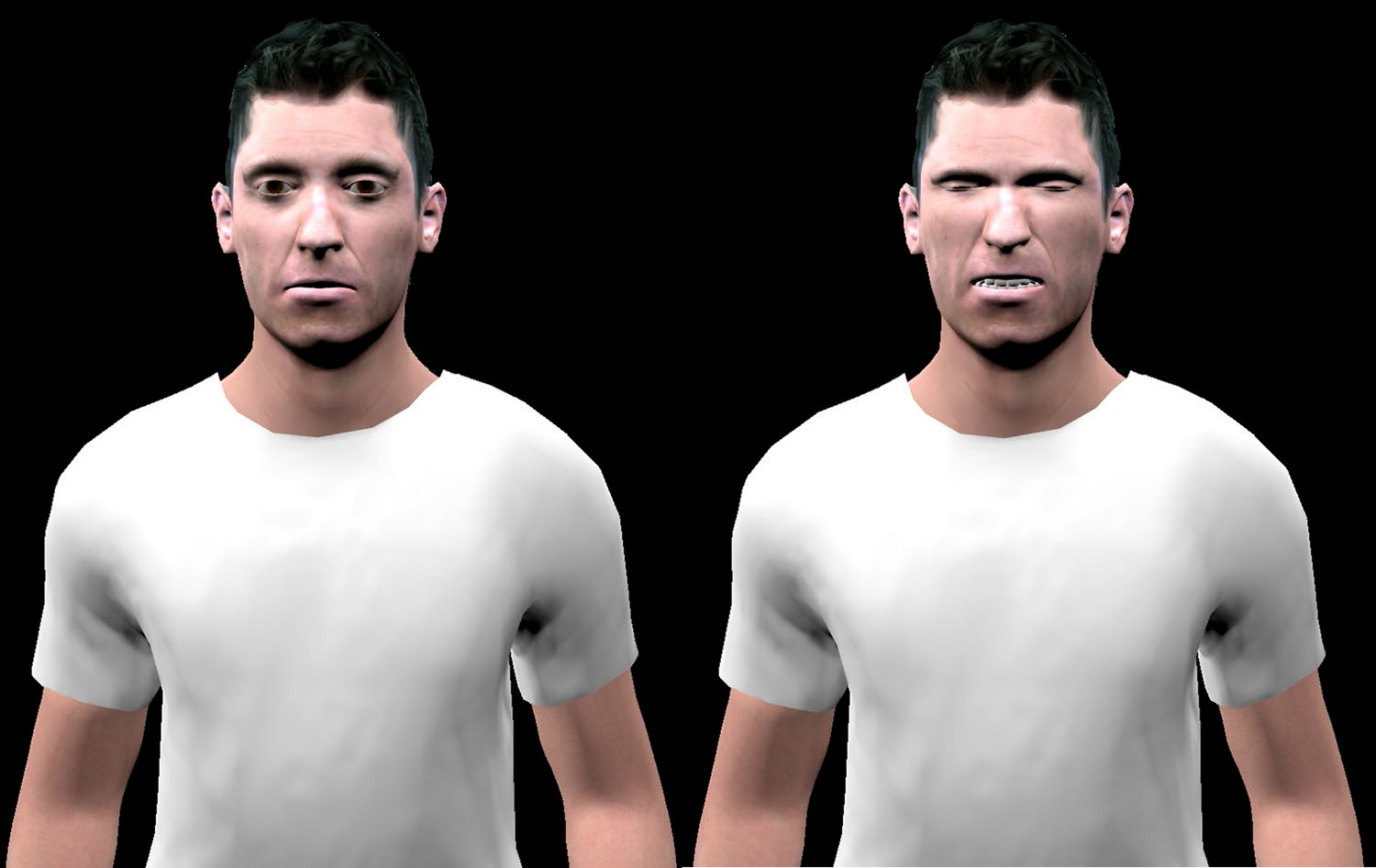
Left side: virtual character at the beginning of the clip (neutral expression). Right side: virtual character expressing pain ( AUs at 60% of maximum contraction) (Fig. 1 was drawn using Blender 2.79 http://www.blender.org).

The stimuli were composed of ten video clips (duration = 4 s, visual only (no audio)) showing the upper body of a male virtual character (Fig. 1) from the Empathy-Enhancing Virtual Evolving Environment (EEVEE)^36^. The video clips were created using Blender (Blender Foundation, Amsterdam, Netherlands). The virtual character was animated with a dynamic facial pain expression at five different intensities (20%, 40%, 60%, 80% and 100%), either with body animation (*idle condition*, see the *Body Animation* section below for a detailed description of the method) or without body animation (*still condition*). The video clips are available online via a link in the Supplementary Material section.

##### Facial pain expression animation

The character’s facial pain expression was created using Action Units (AUs) according to the Facial Action Coding System (FACS)^37^. The characteristic AUs from the pain expression (AU4 (brow lowerer), AU6 (cheek raiser), AU7 (lid tightener), AU9 (nose wrinkler), AU10 (upper lip raiser) and AU43 (eyes closed)); and five occasional AUs (AU12 (lip corner puller), AU20 (lip stretcher), AU25 (lips part), AU26 (jaw drop) and AU27 (mouth stretch))^38^ were manipulated using Blender to create five intensities of facial expression (20%, 40%, 60%, 80% and 100% of their maximum contraction). Each video began with the character showing a neutral expression (AUs set at 0). The level of the AUs were increased linearly to reach the set intensity over one second. The pain expression was then maintained for three seconds until the end of the clip (Fig. 1).

##### Body animation

For the *idle motion condition*, the character’s body was animated with pre-recorded human postural oscillations, i.e., subtle motion specific to dynamic human equilibrium and breathing. For the *still condition*, the character’s body was fixed at the initial posture of the person in the pre-recorded motion capture sequence.

##### Motion capture data

Kinematic data were collected on a young man (age = 20, with anthropometric characteristics similar to the virtual character). He was instructed to stand naturally with his arms alongside his body without moving his feet and looking straight ahead. Data were collected using a Qualisys optoelectronic motion capture system (Göteborg, Sweden) composed of eight infrared cameras sampled at 50 Hz. Markers were placed on the following anatomical landmarks: head of the fifth and first metatarsals, medial and lateral malleolus, medial and lateral femoral condyles, anterior superior iliac spine, zyphoid process at the lower part of the sternum, L5, C7, nasion, acromion process, olecranon, processus styloideus of the ulna and radius^39^. Three more markers were added to record respiratory movements: two on the sternal extremity of the clavicle and one on the navel. The markers’ positions were imported and applied on a skeleton to animate the virtual character using Blender (Blender Foundation, Amsterdam, Netherlands). The corporeal envelope of the character was mapped on the skeleton in Blender and the link between the envelope and the skeleton was set using the “automatic weighted” option available in Blender.

##### Judgement of pain intensity

The task was programmed using Psychopy^40^ and displayed on a computer screen (resolution = 1920 × 1080) at a viewing distance of 70 cm from the participants. Each trial began with the presentation of a video clip (duration = 4 s). After the video clip, the labels “Mild pain” or “Very intense pain” [In French “Douleur peu intense” and “Douleur très intense”] were displayed on each side of the screen. The participants were asked to report the intensity of the virtual character’s pain expression by clicking one of the two labels. Each stimulus was displayed ten times for a total of 100 trials for the task (ten trials * five facial expressions * two body conditions). The order of the stimuli was randomised across the task. After every 20 trials, a pause screen was displayed and the participants clicked on a rectangle in the centre of the screen when they were ready to resume the task. Before the experimental phase, the participants performed a familiarisation phase composed of ten trials presenting each stimulus randomly.

#### Data analysis

The *Very Intense Pain* rating, corresponding to the percentage of “Very Intense Pain” responses, was calculated for each degree of facial pain expression (*20%, 40%, 60%, 80%* and *100%*) in the two body conditions (*still* and *idle*). All statistical analyses were performed using Python libraries (LMFIT^41^ and SciPy^42^).

**Figure 2:**
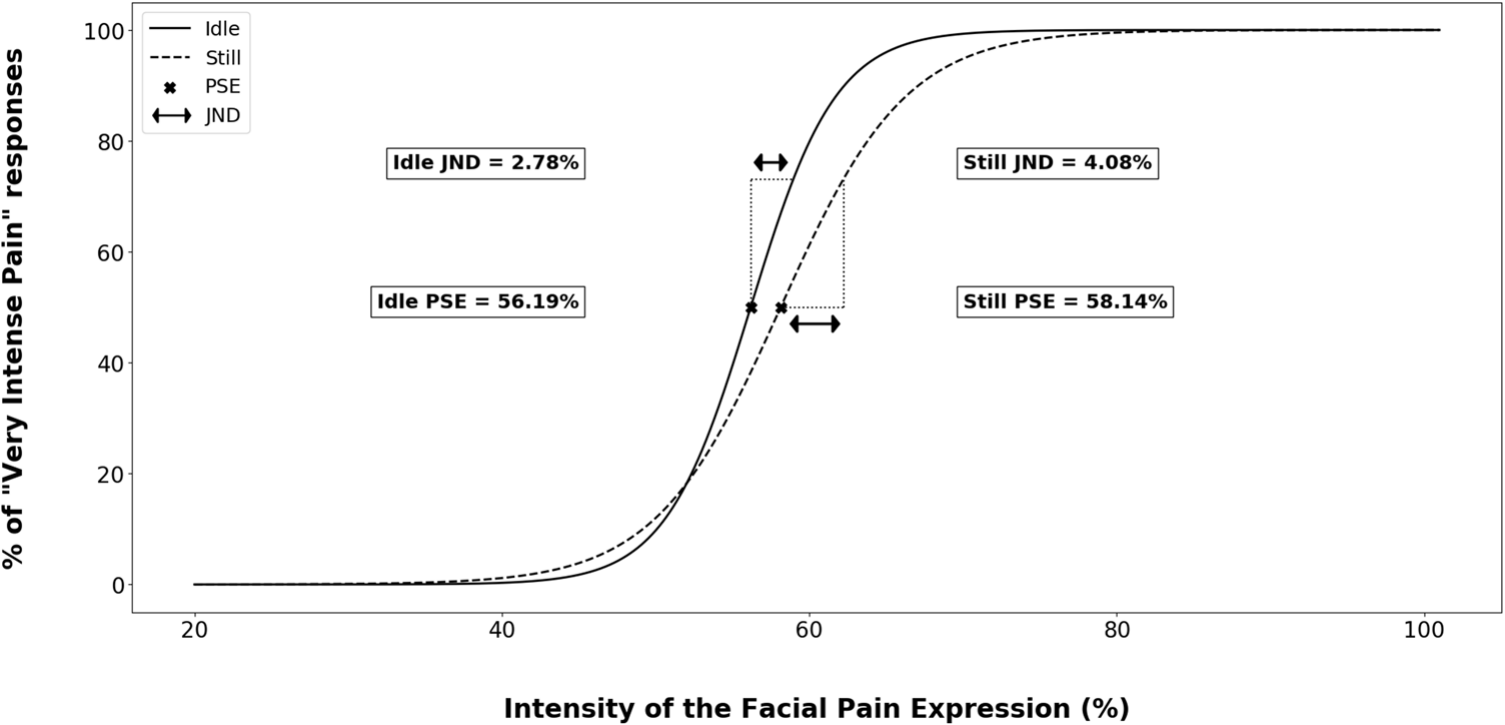
Logistic modelling of “Very Intense Pain” responses according to facial pain expression intensity in the *still* and *idle conditions*. PSE and JND correspond to the mean of PSE and JND obtained from the modelling of each participant.

A logistic function was fitted on each participant’s *Very Intense Pain* rating using the formula: y = 1/(1 + exp(-(x-PSE)/JND)) in order to collect the Point of Subjective Equality (PSE) and the Just Noticeable Difference (JND) (see Fig. 2). The PSE corresponded to the theoretical facial expression intensity at which a participant switched from “Mild Pain” to “Very Intense Pain” (i.e., the intensity of facial expression for which a participant responded “Very Intense Pain” 50% of the time). The JND determined the precision of the categorisation and corresponded to the minimum AU contractions added to the PSE to trigger a quasi-systematic “Very Intense Pain” response. To be included in the analysis, a participant had to produce ratings ranging on average between 0 and 20% for the lowest intensity of pain expression and a rating between 80 and 100% for the most intense expression. Two participants’ ratings were on average 90% and 50% respectively for the lowest intensity of facial pain expression, so they were excluded from analysis. Thus, the final sample for analysis of the psychophysical variables (PSE and JND) included 30 participants. Two-tailed paired t-tests were performed on the PSE and JND to compare the perception of facial pain expression in the *idle* vs. *still* conditions.

### Results

**Figure 3:**
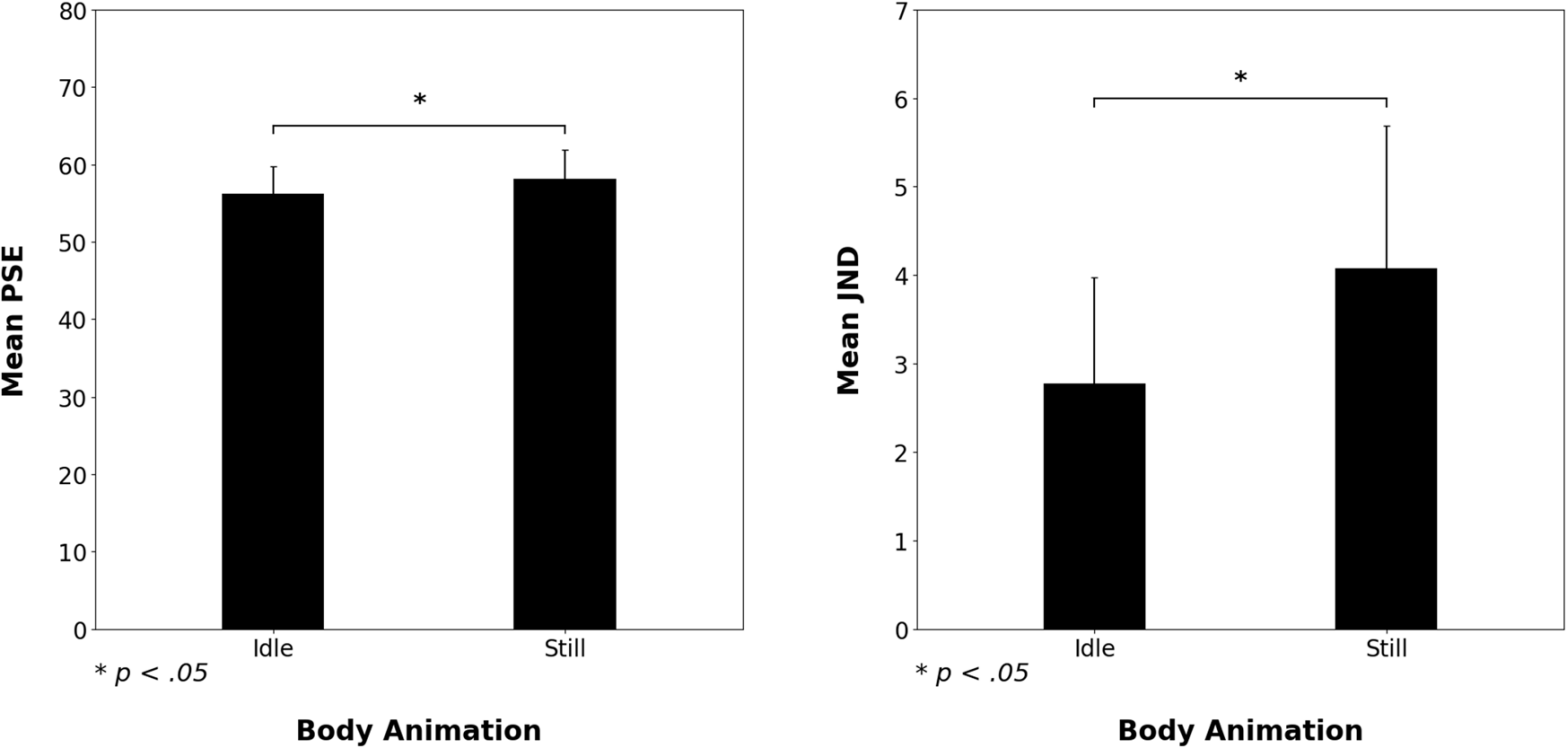
Mean of PSE and JND obtained from the logistic modelling of each participant’s “Very Intense Pain” responses in the *still* and *idle conditions*. Error bars correspond to confidence intervals at 95%.

The two-tailed paired t-test on PSE revealed a significant difference between the *idle* and *still conditions* [t(29) =  − 2.22, *p* = 0.035, *d* = − 0.41]. This result indicated that a lower intensity of facial pain expression was required to be categorised frequently as “Very Intense Pain” in the *idle condition* (PSE mean = 56.19%, 95% CI [52.59, 59.78]) compared with the *still condition* (PSE mean = 58.14%, 95% CI [54.31, 61.97]). The two-tailed paired t-test on the JND also revealed a significant difference between *idle* and *still conditions* [t(29) =  − 2.24, *p* = 0.033, *d* = − 0.42], indicating that the participants were more precise in categorising intensity in the *idle condition* (mean JND = 2.78%, 95% CI [1.57, 3.98]) than in the *still condition* (mean JND = 4.08%, 95% CI [2.46, 5.69]) (see Figs. 2 and 3).

### Summary

The results of this first experiment showed that the presence of idle motion influenced the perceived intensity of the virtual character’s facial pain expression. Indeed, the significantly lower PSE in the *idle condition* compared with the *still condition* indicated that a lower intensity of facial pain expression was necessary to be frequently categorised as “Very Intense Pain” in the presence of postural oscillations compared with the *still condition*. The significantly lower JND in the *idle condition* compared with the *still condition* suggested that a lower intensity of facial expression was needed to reach a quasi-systematic “Very Intense Pain” response threshold in the presence of postural oscillations.

These results showed that the perceived intensity of the virtual character’s pain was modulated by the presence of postural oscillations, compared with a *still condition*. Since these movements are non-communicative, it is likely that natural postural oscillations increased behavioural realism by creating the illusion that the virtual character is alive, which may increase its anthropomorphism and favour perception of its pain expression.

## Experiment 2

Experiment 2 aimed to determine whether adding natural postural oscillations to the virtual character might enhance affective reaction when considering its pain expression. This construct reflects mixed emotions or feelings including self-oriented (e.g., distress) or other-oriented affective responses (e.g., compassion, sympathy)^26,27^ that can stem from empathy for pain in others. Such an affective reaction may vary in response to the intensity of other’s pain expression, and depends on individual factors (e.g., individual capacity of emotion regulation)^22^. Concretely, affective reaction was evaluated in Experiment 2 by asking the participants to report whether they felt affectively touched (In French “Touché(e)”) when viewing pain in the virtual character. It was assumed that the participants would have a greater subjective sense of *being touched* by the virtual character’s pain in the *idle condition* compared with the *still condition*.

### Method

#### Participants

A different group of 34 young adults from the Faculty of Sport Science of Paris-Saclay University took part in the experiment (mean age = 18.56 years, SD = 1.35, range 18–25 years, 17 women). The sample size was based on a previous study with a similar design^10^. Every participant had normal or corrected-to-normal vision. The study was approved by the Ethics Committee of Paris-Saclay University (#CER-Paris-Saclay-2020-147) and the participants gave written informed consent to take part in this study. The experiment was conducted in accordance with the Declaration of Helsinki.

#### Material and tasks

##### Self-report of affect sharing

The task design was the same as in Experiment 1 using the same video clips. Following the video clip, the labels “Not Touched” or “Touched” [In French “Pas Touché(e)” and “Touché(e)”] were presented on either side of the screen. The participants were asked to report whether they were touched by the virtual character’s pain by clicking on one of the two labels. Each stimulus was displayed ten times for a total of 100 trials for the task (ten trials * five facial expressions * two body conditions). The order of stimuli was randomised across the task. Every 20 trials, a pause screen was displayed and the participants clicked on a rectangle in the centre of the screen to resume the task. Before the experimental phase, the participants performed a familiarisation phase composed of ten trials presenting each stimulus in random order.

#### Data analysis

The *Sense of Being Touched* rating, corresponding to the percentage of “Touched” responses, was calculated for each degree of facial pain expression (*20%, 40%, 60%, 80%* and *100%*) in the two body conditions (*still* and *idle*). All statistical analyses were performed using Python libraries (LMFIT^41^ and SciPy^42^).

A logistic function was fitted on the *Sense of Being Touched* rating for each participant using the same formula as in Experiment 1: y = 1/(1 + exp(-(x-PSE)/JND)) in order to collect the Point of Subjective Equality (PSE) and the Just Noticeable Difference (JND) (see Fig. 2 and Experiment 1 for details about this indices). Four participants were excluded from analysis because their ratings did not meet the required assumptions for a logistic function (see Method section of Experiment 1). Thus, the final sample for the analysis on the psychophysical variables (PSE and JND) included 30 participants. Two-tailed paired t-tests were performed on PSE and JND to compare the perception of facial pain expression in *idle* vs. *still conditions*.

### Results

**Figure 4:**
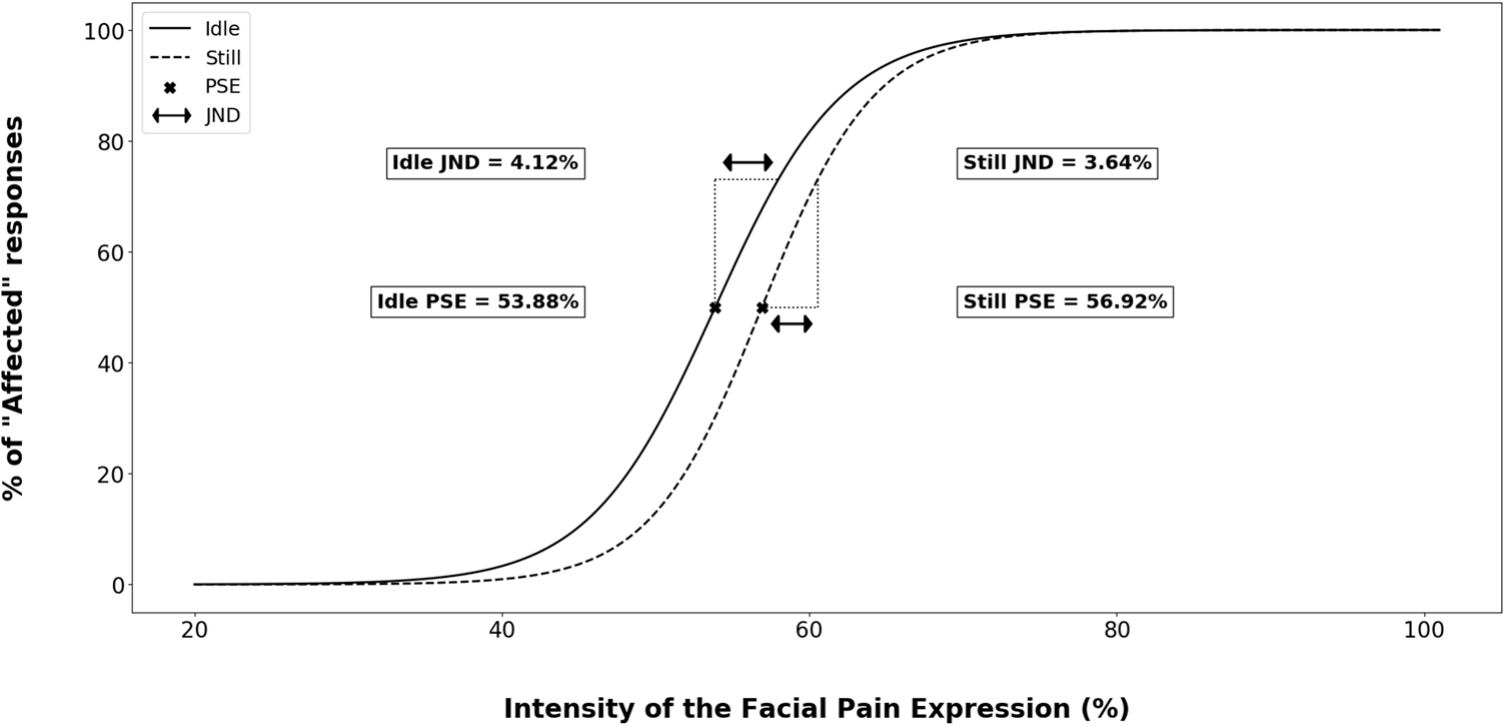
Logistic modelling of “Touched” responses according to facial pain expression intensity in the *still* and *idle conditions*. PSE and JND correspond to the mean of PSE and JND obtained from the modelling of each participant.

**Figure 5:**
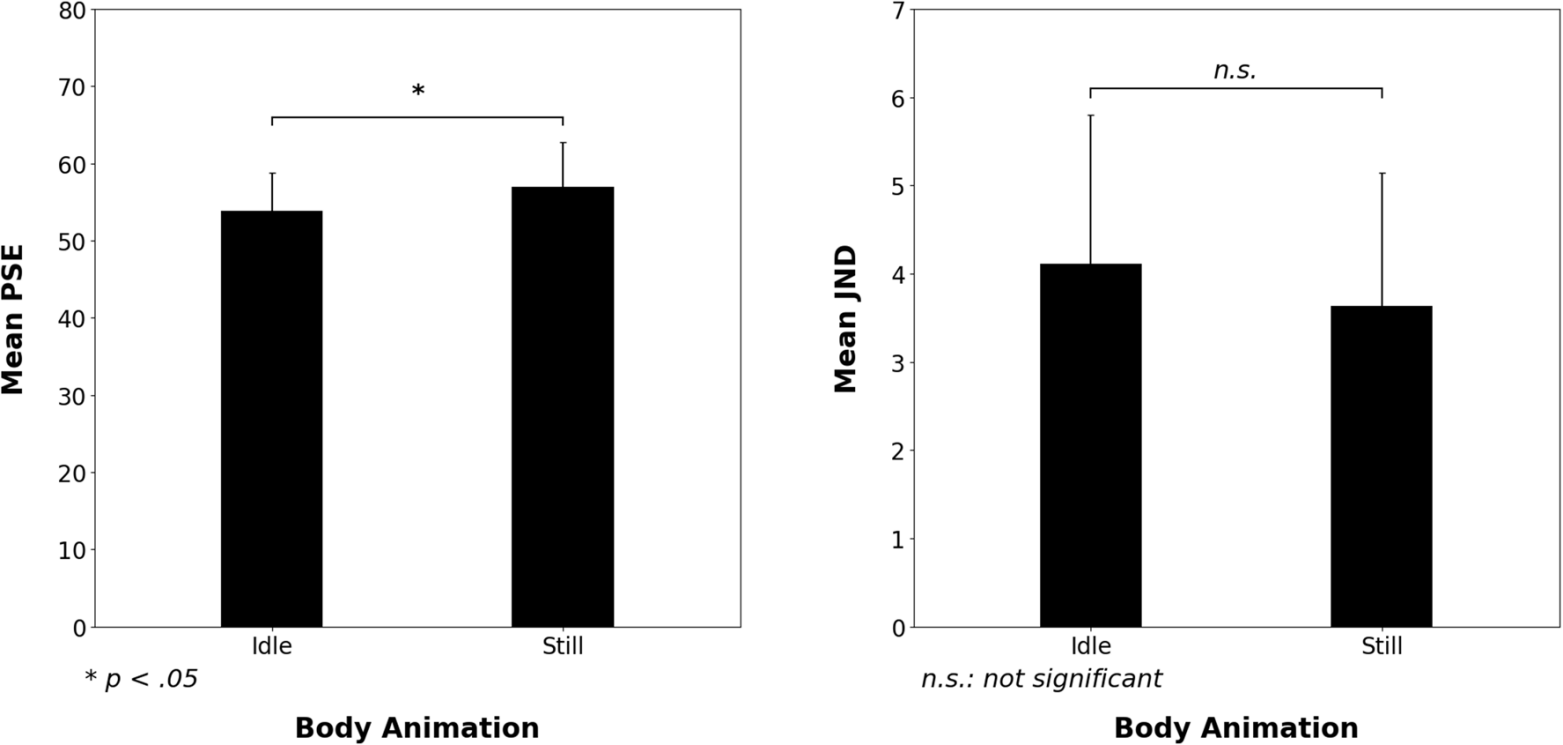
Mean of PSE and JND obtained from the logistic modelling of “Touched” responses of each participant in the *still* and *idle conditions*. Error bars correspond to the confidence intervals at 95%.

The two-tailed paired t test on PSE revealed a significant difference between the *idle* and *still conditions* [t(29) = − 2.15 ; *p* = 0.040, *d* = − 0.40], which indicated that the participants reported being “Touched” by the pain expression at a lower intensity of facial expression in the *idle condition* (PSE mean = 53.9%, 95% CI [48.9%, 58.8%]) than in the *still condition* (PSE mean = 56.9%, 95% CI [51.0%, 62.8%]). The two-tailed paired t-test on JND did not reveal a significant difference between *idle* (mean JND = 4.1%, 95% CI [2.4%, 5.8%]) and *still* (mean JND = 3.6%, 95% CI [2.1%, 5.2%]) *conditions* [t(29) = 0.55 ; *p* = 0.59, *d* = 0.10] (see Figs. 4 and 5).

### Summary

The results showed that the participants were aroused more by the virtual character’s pain expression in the presence of idle motion (natural postural oscillations) than in the *still condition*. Indeed, the PSE was significantly lower in the *idle condition* than in the *still condition*, indicating that a lower intensity of facial pain expression was necessary to touch participants in the *idle condition* than in the *still condition*. By contrast, the JND did not differ between the *idle* and *still conditions*.

In agreement with Experiment 1, it is likely that the presence of postural oscillations made the virtual character seem more human and its emotion more genuine. Thus, the participants would be more sensitive to its pain expression, as reflected by their enhanced empathic response. They would identify its pain as being more intense and would share more in its distress.

## General discussion

The present study revealed the potential of natural postural oscillations to increase behavioural realism in a virtual character, resulting in an increased empathic response towards its facial pain expression. Indeed, the participants perceived its pain as more intense (Experiment 1) and reported being more touched (Experiment 2) when seeing its facial pain expression in the presence of natural postural oscillations compared with the *still condition*. These results confirm previous outcomes obtained by Treal and collaborators^10^, who showed that participants perceived the virtual character’s facial pain expression as more intense and more believable when its trunk was animated with a subtle mechanical oscillatory movement. In the same vein, some studies on robots revealed that robots’ gestures during idle moments (i.e., moments when the robot is not performing a task) may enhance its anthropomorphism^43^ or ‘friendliness’^44^.

The originality of the present work was the use of human motion-captured postural oscillations, based on the bulk of evidence showing that the human visual system is particularly sensitive to biological motion, that would be detected in an early and pre-attentive stage^45^ (for a review and a recent theoretical model). The social function of biological motion in the context of voluntary action is well documented^46–50^. To our knowledge, this is the first study to demonstrate the contribution of postural oscillations in emotion perception research when kinematics contain no socially relevant information (i.e., automatic movements like postural oscillations). Thus, the presence of biological motion in virtual characters might provoke an early unconscious identification of the virtual agent as a natural object (i.e., a human) rather than an artificial one (i.e., a cartoon). This early, pre-attentive identification would significantly influence the latter stages of facial pain expression processing, and ultimately the believability of its pain experience, suggesting a higher empathic response to its pain. This view is in agreement with a recent hierarchical model of social perception^51^ supporting the idea that different sources of visual information would be initially processed and then integrated in later stages of the visual system to shape a whole-person percept that would be consciously experienced. In other words, natural postural oscillations might be far from being a trivial component to trigger a more spontaneous emotional reaction compared with a still condition, which in turn will favour a greater empathic response towards the pain expression of a virtual character. Herein, there was no direct comparison between virtual characters animated with natural postural oscillations versus mechanical idle motion. However, recent studies^34,52^ have shown differences in emotional responses from participants when observing an agent moving naturally or mechanically. Piwek et al.^34^ asked participants to judge the acceptability of different types of virtual characters animated with biological motion or with the same motion distorted at different levels (mechanical motion). They showed that the implementation of biological motion enhanced the characters’ acceptability compared with mechanical motion. Williams et al.^52^ showed that a human body moving naturally elicited both greater visual attentional engagement and autonomic arousal (assessed by pupil size changes) compared with a human body moving mechanically. Thus, it would be interesting to examine whether idle motion implemented naturally rather than mechanically would be a better method to elicit a ‘*suspension of disbelief*’, a term coined by de Gelder and collaborators^2^ to describe acceptance that a virtual character might feel emotion despite knowing it is virtual.

Based on the theoretical models of empathy establishing a clear difference in timing and networks between its components^19,53^, it is proposed that the increased empathic response when the virtual human is animated with natural postural oscillations might be due to a greater affective resonance provoked by its facial pain expression, rather than stemming from a modulation of the cognitive component (i.e., perspective taking) of empathy. Indeed, in a seminal electroencephalography study, Fan and Han^54^ investigated the temporal dynamics of neural activity underlying empathy processes using the event-related potential (ERP) technique. Participants were shown real pictures or cartoons of hands in painful situations. The results revealed that only the early brain response reflecting affective resonance was modulated by the type of painful hand stimuli, as indicated by a later early affective response evoked by painful cartoons compared with human pictures of painful hands. In the same vein, a recent study using fMRI showed that dynamic fear-expressing humans activated stronger brain regions associated with affective resonance (i.e., ventral anterior and posterior cingulate gyrus, the anterior insula and the inferior frontal gyrus) compared with similar face expressions in virtual characters^55^. These works support the idea that the humanness of a virtual character would affect the mechanism of affective resonance more specifically. Nevertheless, it cannot be excluded that the impact of idle motion might be simply the outcome of a perceptual bias. Namely, the mere presence of motion, regardless of its nature, might affect the participant’s subjective judgment in general, and not specifically the ability to empathise with the virtual character’s pain. Then, future investigations using neuroimaging methods and including control conditions (in particular a direct comparison with mechanical idle motion) are needed to explore these theoretical proposals, i.e., the idea of an unconscious primary categorisation of an animated agent as human (i.e., natural object) resulting in greater affective resonance when empathising with its pain, due to the presence of natural postural oscillations. Finally, the present study used only a male character, while it is evidenced that the perception of pain in others can be modulated by the gender of the person in pain^56^. So, it would be of interest to examine whether the potential of natural postural oscillations to enhance the genuineness of a virtual human’s facial pain expression would be modulated by the gender of a virtual character relative to the gender of the participant.

## Conclusion

The use of highly realistic virtual characters to study emotion perception and social cognition offers the possibility to create an interactive, yet well-controlled interaction. Idle motion appears to be a key component of behavioural realism. Surprisingly, there is no experimental data supporting this consensual stance, except for one recent study^10^ and the present work that specifically demonstrated the potential of natural postural oscillations to enhance the empathic response towards pain expressed by a virtual character compared with a still condition. It was argued that human motion capture would be particularly relevant for idle movement animation, which is by essence a ‘by default component’, not designed to be interactive and flexible according to the user’s responses. Moreover, the positive influence of idle motion on the perception of a virtual character’s facial expression needs to be validated with other emotions (e.g., joy, disgust, fear, surprise, etc.) and demonstrated in more complex tasks including contextual and situational information (e.g., a narrative item before stimuli presentation, a contextual background or even more complex scenarios). As stated by scholars in affective neuroscience, the effect of context on the perception of others’ emotion is piecemeal and not sufficiently accounted for in the scientific literature^57^. In the same vein, some authors in affective computing are using virtual settings (i.e., video games) in fundamental research, showing that participants rely on contextual information even more than facial expression to judge a virtual character’s emotion^58,59^. Overall, further research will help elucidate the contribution of idle motion when perceiving emotion in a virtual agent, and more generally when interacting with it.

## Supplementary information

Supplementary information 1.

Supplementary information 2.

Supplementary information 3.

Supplementary information 4.

Supplementary information 5.

Supplementary information 6.

Supplementary information 7.

Supplementary information 8.

Supplementary information 9.

Supplementary information 10.

Supplementary information 11.

## Data Availability

The datasets generated during and/or analyzed during the current study are available from the corresponding author on reasonable request.
