# Supplementary material for: Natural human postural oscillations enhance the empathic response to a facial pain expression in a virtual character
Source: Sci Rep. 2021 Jun 14;11:12493. doi: 10.1038/s41598-021-91710-5 (PMC8203793; doi:10.1038/s41598-021-91710-5)
Supplement: Supplementary file 1 — Supplementary information 1. [file 41598_2021_91710_MOESM1_ESM.docx]

Video idle_20.avi - Virtual character expressing pain at 20% of the maximum contraction of the AUs in the idle condition. (The video was created using Blender 2.79 <http://www.blender.org>)

Video idle_40.avi - Virtual character expressing pain at 40% of the maximum contraction of the AUs in the idle condition. (The video was created using Blender 2.79 http://www.blender.org)

Video idle_60.avi - Virtual character expressing pain at 60% of the maximum contraction of the AUs in the idle condition. (The video was created using Blender 2.79 <http://www.blender.org>)

Video idle_80.avi - Virtual character expressing pain at 80% of the maximum contraction of the AUs in the idle condition. (The video was created using Blender 2.79 <http://www.blender.org>)

Video idle_100.avi - Virtual character expressing pain at 100% of the maximum contraction of the AUs in the idle condition. (The video was created using Blender 2.79 <http://www.blender.org>)

Video still_20.avi - Virtual character expressing pain at 20% of the maximum contraction of the AUs in the still condition. (The video was created using Blender 2.79 <http://www.blender.org>)

Video still_40.avi - Virtual character expressing pain at 40% of the maximum contraction of the AUs in the still condition. (The video was created using Blender 2.79 <http://www.blender.org>)

Video still_60.avi - Virtual character expressing pain at 60% of the maximum contraction of the AUs in the still condition. (The video was created using Blender 2.79 <http://www.blender.org>)

Video still_80.avi - Virtual character expressing pain at 80% of the maximum contraction of the AUs in the still condition. (The video was created using Blender 2.79 <http://www.blender.org>)

Video still_100.avi - Virtual character expressing pain at 100% of the maximum contraction of the AUs in the still condition. (The video was created using Blender 2.79 <http://www.blender.org>)
